# Climate and landscape changes as driving forces for future range shift in southern populations of the European badger

**DOI:** 10.1038/s41598-019-39713-1

**Published:** 2019-02-28

**Authors:** Luís M. Rosalino, Diana Guedes, Diogo Cabecinha, Ana Serronha, Clara Grilo, Margarida Santos-Reis, Pedro Monterroso, João Carvalho, Carlos Fonseca, Xosé Pardavila, Emílio Virgós, Dário Hipólito

**Affiliations:** 1 Departamento de Biologia & CESAM, Universidade de Aveiro, 3810-193 Aveiro, Portugal; 2 cE3c- Centre for Ecology, Evolution and Environmental Changes, Faculdade de Ciências da Universidade de Lisboa, 1749-016 Lisbon, Portugal; 3 CIBIO/InBIO, Centro de Investigação em Biodiversidade e Recursos Genéticos, Universidade do Porto, Campus Agrário de Vairão, 4485-661 Vairão, Portugal; 4 Wildlife Ecology & Health group (WE&H) and Servei d’Ecopatologia de Fauna Salvatge (SEFaS), Departament de Medicina i Cirurgia Animals, Universitat Autònoma de Barcelona, 08193 Bellaterra, Barcelona, Spain; 5 Área de Ecoloxía, Departamento de Bioloxía Funcional, Universidade de Santiago de Compostela, Facultade de Bioloxía - Rúa Lope Gómez de Marzoa, s/n. Campus sur., 15782 Santiago de Compostela, Spain; 6 ESCET, Departamento de Biología, Geología, Física y Química Inorgánica, Universidad Rey Juan Carlos, C/Tulipán s/n., 28933 Móstoles, Spain

## Abstract

Human-Induced Rapid Environmental Change (HIREC), particularly climate change and habitat conversion, affects species distributions worldwide. Here, we aimed to (i) assess the factors that determine range patterns of European badger (*Meles meles*) at the southwestern edge of their distribution and (ii) forecast the possible impacts of future climate and landcover changes on those patterns. We surveyed 272 cells of 5 × 5 km, to assess badger presence and confirmed its occurrence in 95 cells (35%). Our models estimate that badger’s presence is promoted by the occurrence of herbaceous fields and shrublands (5%–10%), and low proportions of *Eucalyptus* plantations (<~15%). Regions with >50% of podzols and eruptive rocks, higher sheep/goat density (>4 ind/km^2^), an absence of cattle, intermediate precipitation regimes (800–1000 mm*/*year) and mild mean temperatures (15–16 °C) are also more likely to host badgers. We predict a decrease in favourability of southern areas for hosting badgers under forecasted climate and landcover change scenarios, which may lead to a northwards retraction of the species southern distribution limit, but the overall landscape favourability is predicted to slightly increase. The forecasted retraction may affect community functional integrity, as its role in southern ecological networks will be vacant.

## Introduction

The conservation of rear-edge populations (i.e. low latitude populations living on species range limits) is crucial for maintaining species evolutionary potential^1^, but since such populations face different ecological conditions and potentially distinct population dynamics from populations in core distribution areas, conservation measures should be context-specific^2^. Rear-edge populations are often small and fragmented/isolated (e.g.,^3^), more vulnerable^4^, and highly sensitive to climate change^2^. Climate change has been acknowledged as a key driver of change affecting the phenology, invasiveness and range of a wide variety of species^5^. However, recent data showed that climate change is not the only determinant of distribution boundaries for many terrestrial species^6,7^, especially for those inhabiting regions long influenced by human activities, such as Mediterranean landscapes^8,9^.

Drivers of Human-Induced Rapid Environmental Change (HIREC)—namely farming, pollution, harvesting or the introduction of exotic species^10^—affect the fundamental relationships between species and ecosystems worldwide, contributing to population declines and range shifts, and ultimately lead to evolutionary changes^11,12^. Such drivers can have a determinant role in wildlife ecological patterns in Mediterranean landscapes due to the long history of human influence on those landscapes^13^. At a regional/country scale, topography drives finer-scale discrepancies in climate and landscape composition^14^, which influences demographic patterns and ecological processes (e.g. survival, competition) that can be especially important at species’ distribution edges. Thus, in a changing world it is increasingly important to understand how species distributions change in response to different types of HIREC factors. This is particularly important in biodiversity hotspots where the scenarios of climate change and socio-economic modifications of the landscapes are especially acute, such as in the Mediterranean^15,16^. Decreased rainfall and higher temperatures are forecasted for this region, with resulting higher levels of aridity^17,18^. Together with land abandonment and woodland encroachment onto grasslands^18^ that impose landcover changes throughout the region, climate change will create new challenges for wildlife inhabiting the Mediterranean that need to be anticipated^19^.

Iberian populations of the European badger (*Meles meles*) constitute a good model for assessing the combined effects of different types of HIREC on edge populations in changing landscapes, since Iberia is currently affected by multifactorial global change^18^ and represents the southwestern extent of the species range^20^. Badger demography and foraging patterns are strongly linked to climate- and agro-ecosystem-induced variations in food availability^21,22^. Climate change and extreme weather events can induce seasonal food shortages that influence reproductive success (e.g. cub survival^22^).

At the core of the European badger’s range, its distribution is mostly constrained by disturbance factors (e.g. Netherlands^23^), such as human population density at lower altitudes and lack of vegetation cover at higher grounds in Moravia (Czech Republic) (e.g.^24^), while in England higher altitudes and afforested areas, especially shrub heath and heather moorland restrict its distribution (e.g.,^25^). However, in its northwestern range (Scotland), badger occurrence is mostly affected by an interaction between climatic and topographic factors (minimum winter temperature) that influences the type of land use (e.g. presence of agricultural patches), as well as by human disturbance (e.g. distance to settlements^26^). In the northernmost part of its range (Finland), badger distribution is limited by climatic conditions that restrict food availability and, consequently, cub survival^27^, as well as by steep topography^28^. Conversely, previous studies suggest that badger distribution in its southwestern limit is constrained by dry and closed landscapes, often linked to low food availability in such areas (e.g. earthworms^29^), and availability of suitable sites for setts^30,31^. Western Mediterranean regions are considered poor badger habitats due to spatio-temporal inconstancies in environmental conditions (especially in drier areas) and resource availability^29,32^, which will be exacerbated by the forecasted increased aridity for this region^33^. This variability in environmental conditions, together with the predicted changes in climate and landcover^18^, raise the need to assess how the rear-edge badger population inhabiting the most southwestern corner of its range may be distributed in future decades. This is critical information to understand badger resilience, guide possible management actions to maintain population connectivity, and ultimately comprehend how ecological processes may be affected in regions where this predator’s distribution may shift.

Based on the variety of factors already recognized as crucial in determining badger distributions throughout its range (see above), we aimed to evaluate which type of HIREC factors might be the most influential drivers determining badger distribution in its southwestern range. Our results allowed us to hypothesize how future climate and/or landcover changes envisaged for this region may impact population range. Using a Species Distribution Model approach (SDM^34^), we first tested five hypotheses regarding drivers of badger distribution in Portugal, i.e. if its range is determined by: (H1) Landcover composition (e.g. afforested environments and highly human-modified landcover have a negative effect^25,35^); (H2) Anthropogenic disturbance (i.e. a high prevalence of human-associated disturbance factors constrain badger distribution^23,24^); (H3) Topography and geology (i.e. higher altitudes associated with colder and less vegetated areas, as well as harder rocks making it more difficult to dig setts, limit badger range^25,36^); and (H4) Climate (i.e. climate variations restrict food availability and, therefore, badger distribution^27,29^). Due to the spatial and temporal environmental complexity and heterogeneity of the Mediterranean biogeographical region^13^, which encompasses most of the Portuguese mainland, badger distribution patterns might not be determined by a unifactorial mechanism but by cumulative effects of diverse factors. Therefore, we also considered a fifth hypothesis (H5) representing the combined effects of landcover, anthropogenic disturbance, and abiotic and climatic factors. Finally, we identified the most favorable regions for badgers in Portugal and predicted the evolution of the badger’s range in the country based on the factors we identified as most influential in determining its current distribution.

## Results

**Figure 1:**
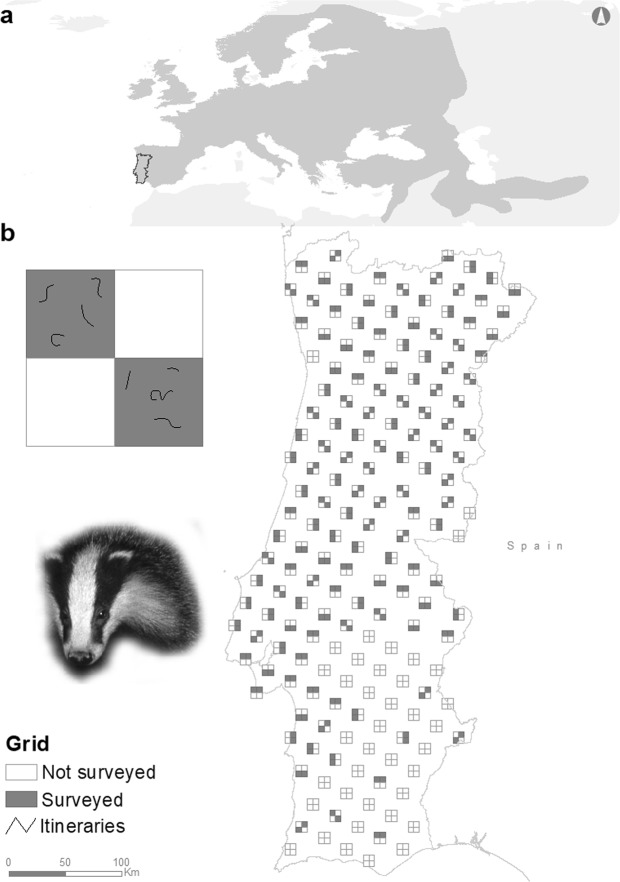
Study area within the distributional range of European badger and the geographical distribution of sampled cells (10 × 10 km and 5 × 5 km; Badger photo: LMR).

We surveyed a total of 1315 transects, representing *ca*. 657.5 km of walked trails, in 272 cells of 5 × 5 km (Fig. 1). Signs of badgers were detected in 95 cells of 5 × 5 km (Fig. S1 - Supplementary Material), which exhibited significant spatial autocorrelation (Moran I = 0.057, *p* < 0.01). We only detected collinearity in one ecogeographical variable included in the topography and geology hypothesis (Tables S1–S4 - Supplementary Material), which was excluded from subsequent analyses: Sediment (Table S3 - Supplementary Material).

The four RAC-BRT (Residuals AutoCovariate Boosted Regression Trees models) models produced for the H1–H4 hypotheses showed that the percentage cover of herbaceous vegetation, shrubland and *Eucalyptus* (H1); the density of goats and sheep and cattle (H2); the percentage of soils dominated by podzols and eruptive rocks (H3); and the annual mean temperature and annual precipitation (H4), were the most influential variables in each hypothesis and the only ones included in the first quarter of relative influence per hypothesis (Table 1). For this reason, we used them as candidate variables for the hybrid hypothesis (H5). Comparing the best BRT models for the first four hypotheses (H1–H4), the one based on landcover composition (H1) was the best fitting, reaching AUC values of 0.853 (SE = 0.018) and a percentage of explained deviance of 52.55% (Table 1).

**Table 1:** Boosted Regression Trees (BRT) model results for hypotheses H1–H4 after simplification and incorporation of the Residuals Autocovariate (RAC).

Variables	Relative Influence
***H1 – Landcover composition (AUC: 0.853*** ± ***0.018)***	
RAC	60.17
**Herbaceous**	**10.78**
**Eucalyptus**	**7.90**
**Shrublands**	**7.16**
H	6.30
Wetlands	4.45
Deciduous	3.24
Food; Agroforestry; Coniferous; Artificial; Exotic	Removed*
***H2 – Anthropogenic disturbance (AUC*** = ***0.833*** ± ***0.029)***	
RAC	70.16
**Goat&sheep**	**15.78**
**Cattle**	**14.06**
Pigs	9.81
Highways; Roads; Unpaved_roads; Human_pop; PA; Hunting	Removed*
***H3 – Environmental abiotic factors (AUC*** = ***0.834*** ± ***0.022)***	
RAC	59.31
**Podzols**	**10.42**
**Eruptive**	**9.32**
Alt_mean	8.45
Alt_range	7.04
Cambisols	5.46
Sediment; Sediment/Metamorph; Luvisols; Lithosols	Removed*
***H4 – Climate (AUC*** = ***0.814*** ± ***0.034)***	
RAC	73.79
**Ann_Temp**	**8.75**
**Ann_Prec**	**8.57**
Prec_season	8.11
Temp_season	0.77

Variables in bold were selected for inclusion in the hybrid hypothesis (H5) RAC-BRT model (i.e. variables included in the two first quarters of relative influence for each hypothesis, when the autocorrelation correction factor is excluded). [*variables removed due to model simplification; Area Under the Curve (AUC ± SE); see Table 2 for definition of variable acronyms].

**Figure 2:**
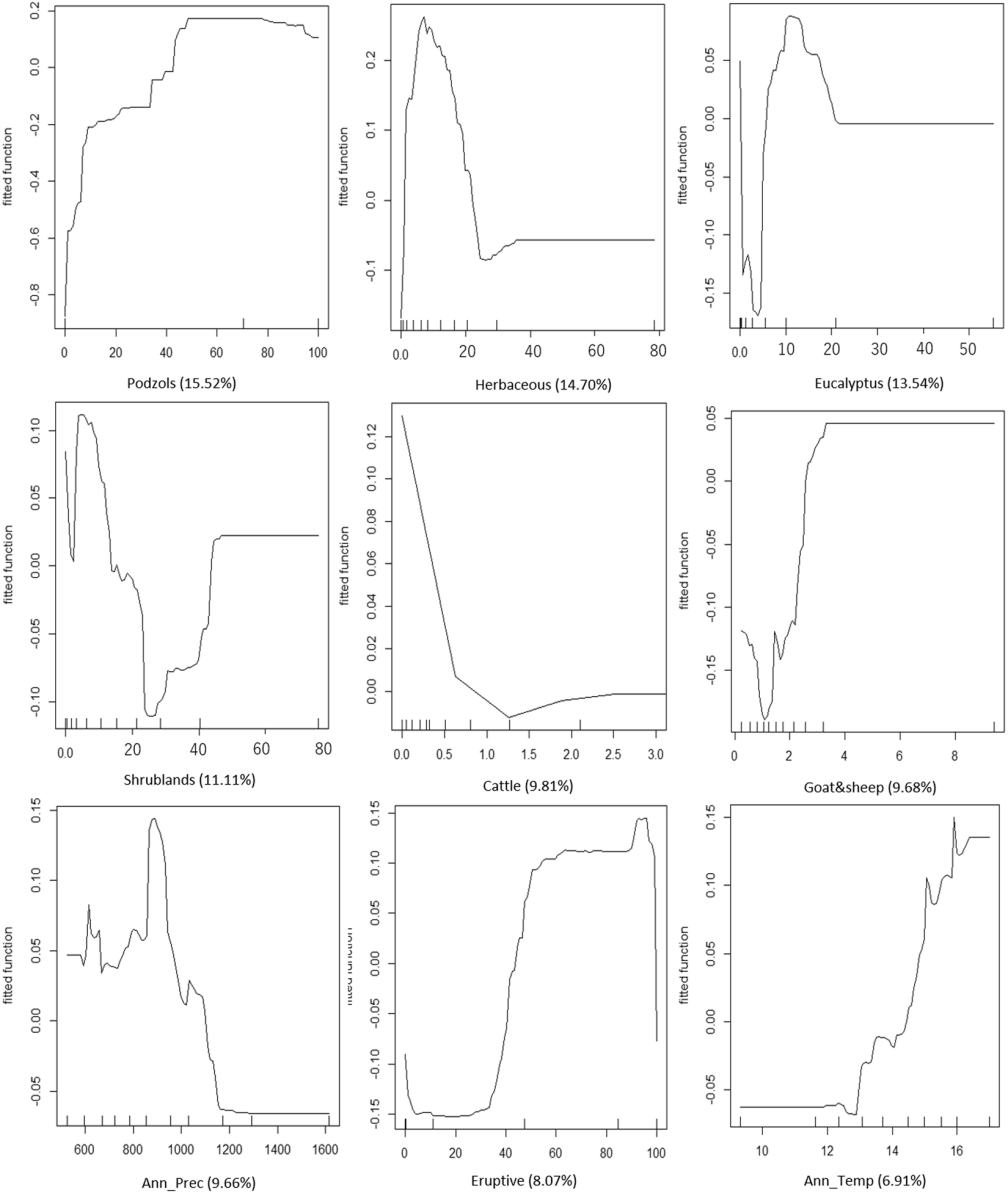
Partial dependence of badger presence on each variable (Y-axis – Model fitted values; X-axis – variables values variation; Herbaceous, Eucalyptus and Shrubland – Percentage of area; Cattle and Goats&sheep - ind./km^2^; Ann_Prec – mm; Podzols and Eruptive - % of area; Ann_Temp - °C); Values within parenthesis represent variable´s relative importance in the final model). Badger predicted distribution is mostly determined by: (1) a low proportion of herbaceous fields, shrublands and *Eucalyptus* cover; (2) high proportions of podzols in the soil structure and eruptive rocks; (3) higher sheep/goat density but lower density/absence of cattle; as well as (4) intermediate rain regimes and mild annual mean temperatures.

We found no multicollinearity between the candidate variables for the hybrid hypothesis (H5) RAC-BRT model, so all of them were used in the modelling procedure (Table S5 - Supplementary Material). The generated model presented a better fit (AUC = 0.858; 44.36% explained deviance) than all those from the previous four hypotheses, indicating that hypothesis H5 was the most supported by our dataset. The differences in AUC values between the H5 model and the other best models produced for hypotheses H1–H4 are not high, but they indicate that the hypotheses are not completely mutually exclusive as H1–H4 included variables also present in the H5 best model. This H5 model highlighted that the percentages of podzol soils and herbaceous landcover were the most influential variables (8.53% and 8.08%, respectively; Fig. 2), with a difference of importance between both of only 5.28% (i.e. a difference of 0.45% represents 5.28% of 8.53%; Fig. 2). The difference between the relative importance of these two variables and other variables is not high, but the difference in percentage of importance reaches ~9% between the second and third most important variables (7.4%, 0.70% difference relative to % importance of podzols; Fig. 2). Furthermore, although the RAC autocovariate has a high relative importance (45.06%), the RAC-BRT model still allows for the environmental and anthropogenic disturbance variables to contribute to deviance reduction of the model while removing the model residuals responsible for spatial autocorrelation^37^. The RAC-BRT model residuals confirm this result, as no significant spatial autocorrelation was detected in the model residuals (Moran I = −0.017, p = 0.067).

The variables included in the best model showed distinct patterns regarding their relationships with badger presence (Fig. 2). Badgers have a higher probability of being present: (1) in areas with herbaceous fields and shrublands, but covering less than 20% and 15% of the landscape, respectively (ideally between 5 and 10% for both landcover types), and where *Eucalyptus* plantations represent <15% of the landscape; (2) in regions with more than 50% of podzols in the soil structure and at least 50% of eruptive rocks; (3) where sheep/goat density is above 4 ind/km^2^, but cattle are absent or exist in low densities (<0.5 ind/km^2^); and (4) in areas subjected to annual precipitation of between 800 and 1000 mm, and mild annual mean temperatures of between 15–16 °C (Fig. 2). Badgers avoid areas almost without goats and sheep (<2 ind/km^2^) (Fig. 2).

**Figure 3:**
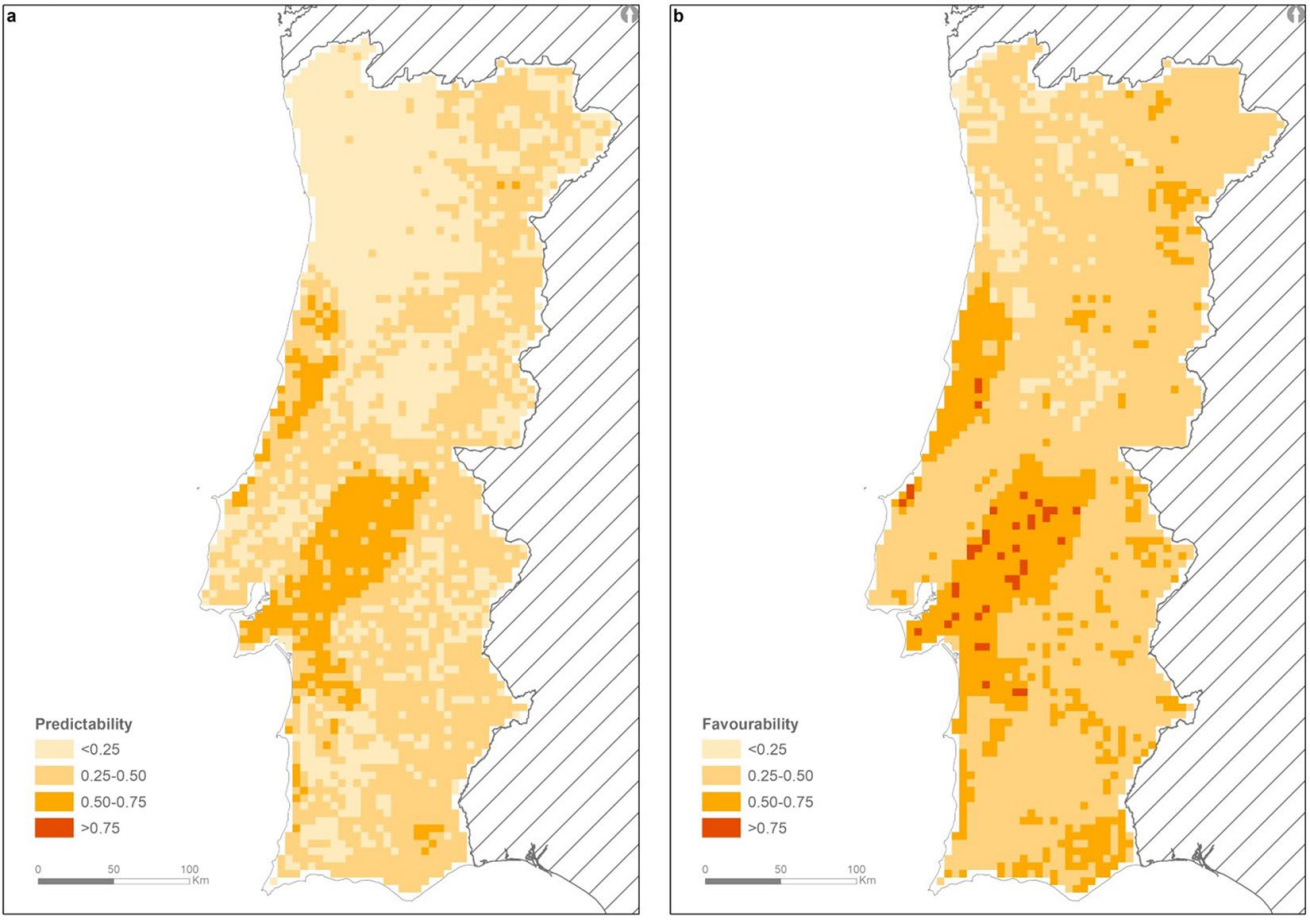
Predictability (**a**) and favourability (**b**) maps of European badger presence in Portugal, showing a central core area where environmental conditions seem more suitable for badgers.

Based on our hybrid model we estimated the probability of badger presence to be high in central Portugal, particularly in the Tejo River basin (a floodplain influenced by this major river) and near the central coast of the country (Fig. 3a). The favourability map confirms this pattern, but also highlighted the importance of many inland regions near the Spanish border (Fig. 3b).

The independent badger presence data (Fig. S2, Supplementary Material) confirmed species presence in 68 of our 5 × 5 km cells. Of those, 44% (N = 30) were identified in our favourability map as moderately or very suitable to host badgers (i.e. F > 0.50). Furthermore, this percentage increased if we considered cells whose favourability almost reached the threshold of F > 0.50; 11 cells had F values in the range 0.45–0.50 (which when included enhanced detection of favourable areas to 60%). The percentage of cells containing independent badger presence data, which were identified by the best model as moderately or very suitable to host badgers (F > 0.45), was similar between areas where monitoring was more representative (Central north; 60.78% of the test cells; Fig. 1) and where sampling was less intensive (Central south; 58.82% of the test cells; Fig. 1).

**Figure 4:**
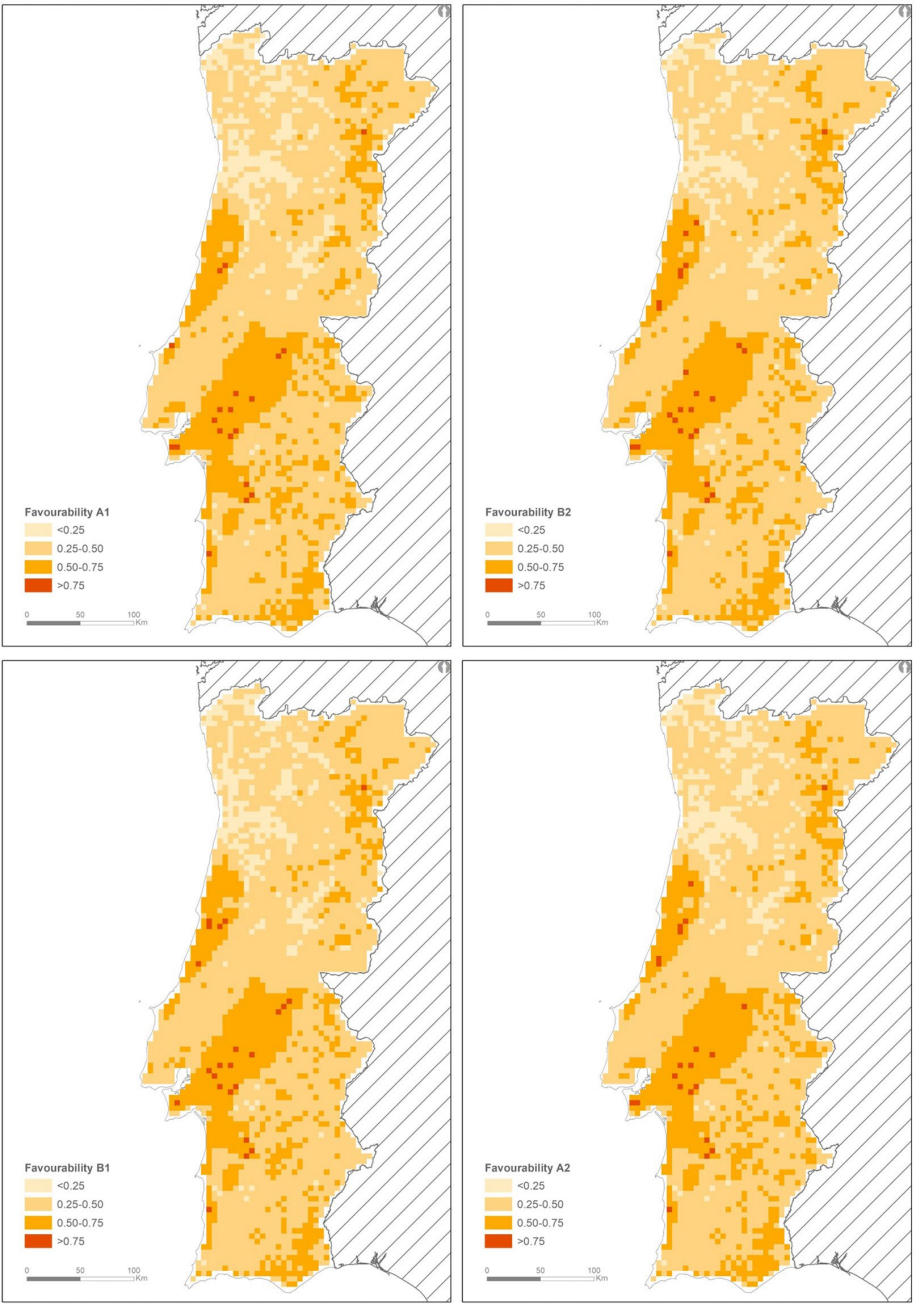
Favourability maps of European badger presence in Portugal estimated for 2040, by applying the best BRT model to different land-use change scenarios, all showing a suitability decrease of the southern edge and an increase in the northeast [Libertarian Europe - A1_2040; Eurosceptic Europe - A2_2040; Social Democracy Europe - B1_2040; European Localism - B2_2040; see^38^ for scenario details] and scenario A1B from the Intergovernmental Panel on Climate Change (IPCC) Special Report on Emission Scenarios (SRES^17^).

We produced favourability maps of badger presence in Portugal for 2040 based on four land-use change scenarios described by Stürck and colleagues^38^ and an IPCC climate prediction (Scenario A1B^17^) (Fig. 4). All scenarios presented a similar output. By 2040, badger landscape favourability seems to increase in the country’s eastern and northeastern regions, but decreases in the North-west. Furthermore, the southern edge of the species range seems to decrease in adequacy for badgers, with a decrease of 50% in the number of cells with favourability >0.75 in area south of the Tejo River (from 35 – current situation - to 16, 15, 17 and 18 for the Libertarian Europe - A1_2040, Eurosceptic Europe - A2_2040, Social Democracy Europe - B1_2040 and European Localism - B2_2040 scenarios, respectively). The central region, which formed a core area of the most adequate territory for the species, loses some regions characterized as highly favourable (red cells), and coverage of cells representing the least suitable areas (lighter orange cells) expands in southern Portugal (evidenced by a slight reduction in this area average favourability, from 0.484 – current situation - to 0.474, 0.473, 0.470 and 0.472 for the A1_2040, A2_2040, B1_2040 and B2_2040 scenarios, respectively). Comparing the overall favourability of the country according to the four forecasted scenarios, we observed a non-significant increase (mean increase: 2.75%; χ^2^ = 1.392, p = 0.707) from the current situation (22.73% of the country showing a favourability >0.5) to favourability percentages of 24.71% (A1_2040), 25.17% (A2_2040), 25.86% (B1_2040) and 26.19% (B2_2040). Thus, the increase in favourability in northwestern areas compensates for the overall decrease in southern regions (Fig. 4)

## Discussion

The estimated current distribution (i.e. range predictability and favourability) of the European badger at the limit of its southwestern range (Portugal) is widespread but patchy, and is mostly determined by a combination of landcover, environmental and anthropogenic disturbance drivers. These drivers are affected by different aspects of HIREC acting synergistically to shape the species’ distribution pattern. Badgers seem to be sensitive to changes in native (herbaceous fields and shrublands) and exotic (*Eucalyptus* plantations) vegetation cover, but also to soil and rock composition (preference for podzols and eruptive rocks), the intensity of pastoral activities (i.e. density of sheep/goats and cattle), and climatic conditions (i.e. temperature and precipitation). This combination of multiple drivers supports our fifth hypothesis (H5).

### Badger distribution

We found that central Portugal is regionally the most favourable area for badgers, with two core critical areas, broadly corresponding to the Tejo River basin and the western coastal plains. Part of the eastern region bordering Spain also has characteristics that favour badger presence (Fig. 3b). This pattern may represent the historical distribution of the species at the southwestern edge of its range. In a study based on badger-associated toponomy (i.e. regional designations or place-names, indicating species presence in the past), Rosalino and colleagues^39^ identified place-names associated with badgers ranging from the south coastal region of Algarve to the northern borders of Portugal, and from the western coast to the eastern regions, near the Spanish border, suggesting that the species was also historically widespread. The patchy favourability for badgers in Portugal reveals that adequate areas for badger survival are discontinuously distributed (Fig. 3b).

### Landscape drivers of badger distribution

Two major habitat factors shape badger distribution in Portugal: landcover and soil/rock structure. Our data indicates that areas showing some heterogeneity are preferred (as also found by Piza-Roca and colleagues^40^). Herbaceous fields and shrublands presented a positive influence on badger presence, up to a threshold of 20% and 15% coverage of the landscape, respectively, with an optimal coverage of between 5 and 10%. Heterogeneous environments can have deleterious effects on some populations, leading to decline or even extinction (e.g. prey species^41^), whereas others can take advantage of the multiple resources they provide. Badgers can benefit from heterogeneous habitats, using the combined resources (food and refuge) provided by such temporal and spatial heterogeneity, especially southern populations (e.g. in semi-arid environments^30,42^; in Mediterranean oak forests^43^). Herbaceous fields can provide easy access to food resources, such as insects (naturally available or derived from use of these patches by domestic ungulates, e.g. dung beetles^44^), earthworms (which are mainly concentrated in herbaceous areas^45,46^), or rodents and rabbits^47,48^. However, badgers are more exposed to humans in such open areas, which are characterized by having less than 20% of cover. Shrublands provide protective cover^43^, but since badgers can be considered more efficient food gatherers than active predators (but see^47^), higher shrub cover makes prey detection harder. Furthermore, high shrub cover is often associated with low abundance of some badger prey, such as earthworms^49^ and rabbits^50^, which may contribute to avoidance of those areas. Therefore, a compromise between protective cover and foraging habitat may have resulted in the selection of areas with low shrubland cover by badgers in Portugal.

*Eucalyptus* plantations cover *ca*. 9% of the Portuguese territory and represent 26% of all Portuguese forested areas^51^. They have been shown to have a negative effect on southern badger populations^35,43,52^. The effects of *Eucalyptus* plantations on wildlife are often associated with food scarcity and high disturbance^35^. However, these habitats can harbour abundant populations of prey if understory shrubs are managed properly (e.g.^53,54^) to provide efficient protective cover, especially in the absence of native forests^55^. Furthermore, their degrees of disturbance vary with harvesting phase (i.e. higher in pre-harvesting phases^56^) and plantation extent^35^. Our results support a positive effect of scarce *Eucalyptus* habitats (<~15% of the landscape), which likely deliver a combination of food resource availability, protective cover, and reduced anthropogenic disturbance.

We found that not only above-ground habitat characteristics drive badger presence in Portugal, since soil and rock types also emerged as being influential in our analysis. Podzols are often formed under forest ecosystems and are usually considered poor soils for agriculture as they possess low levels of moisture and lack many nutrients^57,58^. Although use of agricultural landscapes by badgers may be a mechanism to facilitate access to food (e.g.,^30,43^), agricultural fields suffer frequent soil mobilization to prepare the land for planting or when harvesting production and they are subjected to high human disturbance. This high disturbance level likely limits badger presence, with this latter being more common in areas not suitable for agriculture and where soils are covered by forests, such as those dominated by podzols (>50% of podzols in the soil structure, as detected by our analysis). Often these more “natural” habitat patches are composed of a mixture of tree cover in different successional stages (from forest with sparse understory or shrublands with sparse tree cover to more closed environments), which may provide the necessary cover/protection badgers need. This heterogeneous structure may not have been completely captured by our landcover classes (often composed of monotypic landcover types), preventing a more accurate assessment of their importance in our models. Setts are essential structures for badgers and their stability is a determining factor for population survival^59^. Eruptive rocks can provide such stability, although they also present badgers with a huge challenge when digging a sett. Under such conditions, geological discontinuities may facilitate sett-building^31^. However, no data was available at a national scale to allow us to test this hypothesis.

Apart from *Eucalyptus* plantations, other anthropogenic factors contribute to shaping badger distribution in Portugal. Our results suggest that badgers avoid areas with higher cattle density (>0.5 ind/km^2^), but high densities of sheep/goats (>4 ind/km^2^) promote their presence. Although there is some previous data showing that badgers use agroforestry systems devoted to cattle-raising in Iberia (usually in low density regimes^60^), badgers are also known to avoid cattle in many other regions^61,62^ due to disturbance. In Portugal, sheep and goats are mostly raised in flocks that move around the landscape, perhaps promoting higher concentrations of dung that increase dung beetle availability, a badger prey^48^. Finally, these livestock may also control shrub coverage^63^, which may prevent woodland encroachment onto badger-preferred herbaceous habitat.

### Climatic drivers of badger distribution

Our data showed that climate is an important driver shaping the distribution of rear-edge badger populations. Badgers have a higher probability of using areas with mild climatic characteristics, namely with intermediate precipitation regimes (between 800 and 1000 mm rainfall annually), and mild annual mean temperatures (between 15–16 °C) (Fig. 2).

There are two critical periods for badger survival during their lifetimes, i.e. the first months of life for cubs and over-winter survival for all age classes^22,64^. The first period is mostly affected by extended summer drought, whereas the second is predominantly dependent on winter frost, low temperatures and heavy rainfall leading to floods. In northwestern badger populations, mild winters enhance over-winter survival (e.g.,^64^), as animals (especially juveniles) have a better likelihood of maintaining their body weight and energy reserves. In the UK, Noonan and colleagues^65^ found that badgers reduce their activity (and probably foraging bout length and frequency) when temperatures are lower, jeopardizing their efficiency in accumulating over-winter reserves. Other mesocarnivores and small carnivore species, such as raccoon dogs (*Nyctereutes procyonoides*) or least weasel (*Mustela nivalis*), exhibit similar strategies to cope with critical winter temperatures, due to the high costs of thermoregulation during activity^66,67^. Winter temperatures often drop below 0 °C in many regions of Portugal, perhaps inducing southwestern badger populations to adopt a strategy similar to that of their conspecifics at higher latitudes and reduce their over-winter activity. The biological costs of employing this behavioural strategy are: (i) reduced survival^68^ or (ii) avoidance of areas with particularly harsh winters, i.e. those with lower annual temperatures. Both consequences would result in the same spatial pattern. Our results suggest that lower temperatures are more constraining for Portuguese badger populations than higher ones. Temperatures in Portugal can exceed 35 °C, contributing to mean annual temperatures around 15–16 °C, which we identified as promoting badger presence. Thus, it is possible that badger populations inhabiting this region may have developed local adaptations that allow them to benefit from more temperate conditions. The mechanistic basis for this spatial pattern may also be linked to food resource availability. Milder climates can influence the availability of two of the foods most consumed by badgers in Portugal, i.e. coleopterans and olives^48^. Mild temperatures may increase winter coleopteran survival (e.g.^69^), thereby promoting higher species abundances. Furthermore, the reproductive structures and fruits of olive trees are sensitive to low temperatures, particularly frost^70^, so they present higher productivity in areas less affected by frost.

At this edge of the species distribution, the amount of rain may also be a more important driver of badger presence. Portugal is mostly characterized by a Mediterranean climate where rain mainly falls in winter and cyclic droughts occur^71^, with annual precipitation ranging from <400 mm in southeastern regions to >3000 mm in northwestern ones^71^. Badgers seem to prefer areas with mild rainy conditions (between 800 and 1000 mm of annual precipitation), likely due to the consequently higher availability of some feeding resources (e.g.^72^). Intermediate levels of rainfall mean greater likelihood of avoiding food shortages associated with drought or arid environments^46,73^. Nouvellet and colleagues^68^ showed that cub and juvenile survival were highest under conditions of intermediate levels of rainfall, but adult survival was mostly affected by the driest years, probably due to a decrease in food availability and quality in dry years^64,74^. However, excessive rainfall can affect thermoregulation during winter and early spring, especially when associated with lower temperatures, and it may constrain cub survival during the critical first months outside the sett^68^. Hypothermic stress associated with wetter conditions can debilitate a cub’s immune system, often promoting endoparasitic infections^22^ that may compromise cub survival and recruitment^22,68^. Moreover, depending on the soil and geological structure of the area, high rainfall can also compromise sett stability (e.g. in sandy soils) and habitability (e.g. flooding when sited in valleys or in clay-dominated soils^75,76^). Setts are crucial structures for central-place foragers such as badgers, where animals rest during the day, interact to maintain social cohesion, and where cubs are born and reared^77,78^. Poor sett-building conditions affect species density^79^, especially in Mediterranean areas where sett sites are the main limiting factor constraining distribution^31^.

### Future impact of climate and landcover change on badger distribution

Badgers are central place foragers, whose ecology is intrinsically linked to sett locations^78^. They do not exhibit migratory movements that can lead to changes in their range limits, and so are more vulnerable to climate change^21^. However, badger populations at the northern limit of the species range might benefit from climate change that creates conditions for badgers to colonize environments historically inaccessible to them due to extreme weather (e.g. longer snow-free periods with consequently higher food availability^27,28^). However, at its southern rear-edge range, badgers might experience the inverse pattern due to the combined effects of HIREC factors. As mentioned before, climate change scenarios for Portugal estimate a generalized reduction in annual mean precipitation (by 10% up to 2040^17^) and an increase in temperature (~1.5 °C^17^). This forecasted climate change, together with the predicted changes in landscape composition^38^, will decrease the favourability of areas of southern Portugal for badgers, potentially leading to a range retraction northwards. This retraction will likely be matched by an increase in favourability for northeastern Portugal, where badgers will find better conditions to survive (Fig. 4). Although we forecast a contraction of the badger’s southern range limit in Portugal, we estimate no overall difference in the percentage of areas in the country with higher favourability. Thus, loss of favourable areas in the south will be compensated for by more beneficial environmental conditions in the North-east.

These forecasted changes in distribution for the species in its southwestern rear-edge range can probably occur throughout the badger’s southern distribution range (i.e. the Mediterranean), since the climate change predicted for Portugal is similar to that foreseen for the entire Mediterranean region^17^. Moreover, landscape changes seem likely to follow the same patterns across the region^38^. Nevertheless, further broad-range studies targeting the drivers of distribution of central and eastern Mediterranean badger populations should be prioritized, especially to confirm if the drivers we identified for Portuguese populations have a broader effect and to assess how badger distributions will evolve in those areas. Information covering the entire southern range limit will allow us to understand the ecological strategies badger populations need to adopt to survive in the environmentally challenging landscape of Mediterranean Europe and provide data that can make conservation strategies more effective.

## Methods

### Study area and sampling design

To avoid the limitations associated with studies of local or even regional extent (i.e. calibration of models with limited variation of environmental and anthropogenic disturbance factors affecting model performance and predictability robustness for wider extents^14^), we extended our analysis across all mainland Portugal. This is the first study to evaluate badger distribution and the factors determining it at a nationwide scale in the Mediterranean region. Furthermore, our study encompasses the high bioclimatic variability characteristic of Western Iberia, where Atlantic and Mediterranean biogeographical regions interconnect^80^, as well as high variation in landcover, topography and disturbance conditions.

Western Iberia is characterized by a wide variety of climatic conditions typical of the two distinct bioclimatic regions covering the area: Atlantic (Cantabroatlantic sub-region) and Mediterranean (Sado-Divisorian, Luso-Extremaduran and Carpetano-Leonese sub-regions)^80^. Within the Mediterranean region, the climate is usually hot and dry in summer and humid cool in winter. Heavy rain occurs often, and summer droughts are common and sometimes prolonged^81^. In the Atlantic region, the climate is typically oceanic, with mild temperatures and high precipitation and humidity^82^ (see Table 2 for details). Landcover is diverse, associated with the altitudinal variation (0–1993 m), and includes deciduous (e.g. *Quercus* spp.) and conifer (e.g. *Pinus* spp.) forests and exotic plantations (e.g. *Eucalyptus* sp.), scrublands, natural pastures and agricultural patches (e.g. orchards, olive groves, vineyards, agroforestry, etc.^16^; see Table 2 for details). Mean population density for Portugal is 111.8 inhabitants/km^2^ (2016 data^83^), and the country has a fair coverage of highways (totaling 3,065 km) and 2-lane national, regional and municipal paved roads (totalling 14,313 km) (2016 data; www.pordata.pt/; see Table 2 for details). Cattle, sheep and goats are raised extensively in many regions, reaching average densities of 12.8, 24.0 and 4.4 ind./km^2^, respectively (National Statistics Institute, https://www.ine.pt/; see Table 2 for details).

**Table 2:** Variables used to characterize each 5 × 5 km cell, grouped according to working hypotheses, their mean values and range (corresponding only to those cells that were sampled), and data source (*^91^).

Variable	Description	Mean [range]	Data source
***H1 – Land cover composition***			
Deciduous	Percentage of area covered by deciduous forests, e.g. cork oak forests or olm oak forests.	10.3 [0–62.9]	Land use and landcover map of continental Portugal - COS2007^106^
Coniferous	Percentage of area covered by coniferous forests, e.g. pine forests.	11.3 [0–68.1]	
Agroforestry	Percentage of area covered by agroforestry systems, i.e. agricultural areas under a tree layer.	4.7 [0–71.2]	
Eucalyptus	Percentage of area covered by exotic *Eucalyptus* plantations	6.4 [0–55.3]	
Exotic	Percentage of area covered by other exotic species forests, e.g. acacia or mimosa.	<0.01 [0–2.4]	
Shrublands	Percentage of area covered by shrub or sclerophyllous vegetation.	15.6 [0–75.9]	
Wetlands	Percentage of area covered by rivers, dams, lagoons, marshes or mangroves.	1.1 [0–42.4]	
Herbaceous	Percentage of area covered by herbaceous vegetation, pasture or crops without irrigation.	12.7 [0–78.4]	
Food	Percentage of area covered by food production areas such as vineyards, olive groves, orchards or house gardens.	17 [0–75]	
Artificial	Percentage of area covered by settlements and human-made infrastructure, e.g. urban areas or infrastructures (buildings, bridges).	4.8 [0–56.3]	
H	Shannon–Wiener index*, based on the number and proportion of area occupied by each habitat patch in each cell.	1.55 [0.70–2.10]	
***H2 – Anthropogenic disturbance***			
Highways	Density of 4-lane highways (km/km^2^).	0.08 [0–0.95]	*OpenStreet Map* data^107^
Roads	Density of 2-lane national, regional and municipal paved roads (km/km^2^).	1.63 [0–12.29]	
Unpaved_roads	Density of unpaved roads (km/km^2^).	1.23 [0–6.96]	
Human_pop	Density of human population (ind./km^2^).	165.3 [0–8435.76]	GeoStat databases (Eurostat and the National Statistical Institutes initiative to produce geospatial statistics for EU countries) - http://ec.europa.eu/eurostat/
PA	Percentage of area covered by protected areas.	21.8 [0–100]	Institute for Nature Conservation and Forest (ICNF) - http://www.icnf.pt/
Hunting	Presence of hunting areas.	Binary	
Cattle	Density of cattle (ind./km^2^).	1.25 [<−0.01–62.20]	National Statistics Institute (INE) - https://www.ine.pt/
Goat&sheep	Density of goats and sheep (ind./km^2^).	1.83 [0.25–9.42]	
Pigs	Density of pigs (ind./km^2^).	2.28 [<0.01–129.40]	
***H3 – Environmental abiotic factors***			
Alt_mean	Mean altitude (m).	347.73 [5.84–1297.59]	ASTER Global Digital Elevation Model platform - https://asterweb.jpl.nasa.gov/gdem.asp (Resolution 30 × 30 m)
Alt_range	Altitude range, i.e. difference between maximum and minimum altitude (m).	286.97 [14.00–1350.00]	
Sediment	Percentage of area covered by sedimentary formations.	34 [0–100]	Portuguese Environmental Atlas^108^
Sediment/Metamorph	Percentage of area covered by sedimentary and metamorphic formations.	36.2 [0–100]	
Eruptive	Percentage of area covered by eruptive rocks.	28.6 [0–100]	
Podzols	Percentage of area covered by podzols.	12.4 [0–100]	
Luvisols	Percentage of area covered by luvisols.	13.7 [0–100]	
Lithosols	Percentage of area covered by lithosols.	13.9 [0–100]	
Cambisols	Percentage of area covered by cambisols.	50.2 [0–100]	
***H4 – Climate***			
Ann_Prec	Annual precipitation (mm).	907.93 [526.63–1614.83]	WorldClim – Global Climate Data database (http://www.worldclim.org ^109^; (Resolution 1 × 1 km)
Prec_season	Precipitation seasonality (mm).	55.43 [41.59–67.74]	
Ann_Temp	Annual mean temperature (°C).	14.13 [9.33–17.00]	
Temp_season	Temperature seasonality (°C).	42.83 [30.00–50.77]	

We divided mainland Portugal (89,060 km^2^) into 987 cells of 10 × 10 km, using the UTM reference system, fuse 29, on WGS84 datum (EPSG code: 32629) (Fig. 1). We then selected 180 regularly distributed cells, using the chess knight movement pattern (i.e. L-shaped in any direction), starting at the northwest corner of the country (Fig. 1). Cells located in the L tips were selected for badger sampling and were subdivided into four 5 × 5 km cells, of which we randomly selected two and defined five 500 m line transects/itineraries in each. Transects were not set in areas where badger presence is highly unlikely (e.g. rivers, dams, inside estuaries, beaches) or where much of the landscape is humanized (e.g. villages, industrial compounds). The spatial allocation of sampling transects was defined to proportionally represent, as much as possible, the landscape composition of each 5 × 5 km sampled cell based on landcover characteristics. They were defined manually over a landcover map, within a Geographical Information System, and we tried to correlate the transect length within a specific landcover unit with the approximate proportion of that unit within the sampled cell.

### Survey of badger presence

We surveyed all transects located in the 272 5 × 5 km cells (located in 136 10 × 10 km cells) between June 2014 and January 2017 to detect signs of badger presence. However, 38 of the 10 × 10 km cells, located in the southeastern part of the country, could not be sampled due to logistical limitations (i.e. we could sample ca. 78% of the pre-selected cells; Fig. 1). The remaining six 10 × 10 km cells were not sampled because they encompassed >75% of its area covered by sea, dams or were located within Spanish territory. Badger presence in each transect was confirmed based on signs of species presence, such as footprints, latrines, setts or fur samples found, for example, on barbed wire. Although expert-based identification of carnivore scats is prone to false positive and false negative errors^84,85^, badger scent-marking behaviour minimizes this bias and makes sign identification highly accurate as scats are mostly deposited in ground pits called latrines^78^. Badger setts—underground dens where badgers rest during the day and where their cubs are born and reared^59^—were identified based on the existence of other signs of badger presence in their vicinity and burrow size and structure^59^. When badger presence was confirmed in any transect, the corresponding 5 × 5 km cell was classified as positive.

### Compilation of environmental and anthropogenic disturbance data

Each 5 × 5 km cell was characterized regarding its environmental and anthropogenic disturbance features, mostly based on remote sensing data. We first built a Geographical Information System (GIS) using several software tools (ArcGIS 10.4.1^86^; Quantum GIS 2.14.9^87^) and incorporating the following digital layers: landcover, road and highway network, human density, protected areas and hunting reserves, domestic ungulate densities, topography, soil types, lithology, and climatic data (Table 2).

### Data analysis

We first assessed the spatial autocorrelation of badger presence data in the 5 × 5 km cells and model residuals using the Moran I index^88^, available in the R package “ape”^89^, to prevent poor inference and enhance the predictive ability of the models^90^. We then tested for data multicollinearity between all co-variates using the Variance Inflation Factor (VIF), to identify candidate variables that are collinear^91^. As there is no VIF factual cut off level, we used the values suggested by Zuur and colleagues^92^, and excluded all variables with VIF > 5. The VIF was recalculated for the remaining variables. The process was repeated until none of the retained variables reached VIF > 5.

We tested the factors potentially shaping badger distribution in Portugal using a Boosted Regression Trees (BRT) approach for each hypothesis (H1–H5, see Introduction). BRT combines decision trees and boosting^93,94^. It is based on the average of many prediction rules, achieved in a forward stage-wise procedure (a kind of additive regression model, wherein individual trees act as individual terms^95^), instead of a single-rule prediction. The BRT procedure starts with a regression tree that focuses on minimizing the loss function. Then a new regression tree, containing (or not) variables and nodes distinct from the first tree, is fitted to the prediction residuals of the first tree. At this stage the model comprises two trees, and its residuals are estimated. The overall BRT model is a linear combination of all the generated trees^94^, so global fit is improved by encompassing the predictions of previous trees (weak learners) and focusing on observations incorrectly classified by those trees^93^. We selected this approach because BRT is insensitive to outliers, can fit nonlinear relationships, allows use of different types of variables (e.g. continuous and binary), and automatically models interactions between predictors^94,95^.

Following the recommendations of Elith and colleagues^94^ and Elith and Leathwick^96^, we used a 10-fold cross-validation procedure and selected the largest learning rate (*lr*) and the smallest tree complexity (*tc*) to enable us to achieve a minimum of 1000 trees in the BRT fitting process (see Elith and colleagues^94^ for more details regarding the BRT fitting procedure and *lr* and *tc*). When fitting the consecutive trees, non-informative variables were removed (i.e. the least important variables were excluded and the model was re-fitted in a process we repeated sequentially until no change was achieved in either the % deviance explained or Area Under the Curve - AUC; see below), leading to simplification of the set of variables^94^. The relative contributions of predictors (% importance - the frequency that a variable is selected in the BRT fitting procedure, scaled to sum 100) were calculated, and partial dependence plots were produced for the most important predictors, showing their effects on probability of badger occurrence after accounting for the average effects of other variables. BRT models were fitted using the R-package ‘gbm’^97^.

Since we detected significant spatial autocorrelation among residuals (see Results), we adopted the methodology of Crase and collaborators^37^ to incorporate this spatial structure in our BRT to minimize its effects. We added an autocovariate term into the BRT that already contained the environmental and anthropogenic disturbance variables, which accounted for the influence of neighbouring observations by specifying the relationship between the value of a cell and those located in its vicinity, thereby representing the spatial autocorrelation in the residuals^37^. We produced residuals autocovariate (RAC) models for each hypothesis, incorporating into BRT an autocovariate estimated from the residuals of BRTs produced using only the variables described in Table 2. The BRT procedure was the same as that described above and the RAC was estimated using the R package “raster”^98^.

To test the first four of our pre-defined hypotheses (H1–H4), we produced residuals autocovariate BRT models (RAC-BRT) for each hypothesis by combining all the non-correlated variables related to that hypothesis (see Table 2). We then identified the variables representing the 50% more influential predictors in each hypothesis (H1–H4, excluding the autocorrelation correction factor), based on the variables relative influence (i.e. a percentage representing the average number of times a variable is used to define a split of a tree branch, weighted by the improvement of the model fit due to that split^99^). These variables were used to produce a hybrid hypothesis (H5), which was tested using the same methodological approach. Selection of the best hypothesis explaining the pattern of badger distribution was based on the Area Under the Curve (AUC, derived from the Receiver Operating Characteristic curve, or ROC) of the ensemble RAC-BRT models of each hypothesis^93^. The best model structural fit (i.e. % deviance explained) was also estimated.

Based on our modelling results, we first implemented a model-based interpolation process (i.e. a prediction of species presence in new cells that present a similar range of environmental characteristics to those of the sampled cells within the same time-period evaluated^34^) to estimate badger distribution in the species southwestern range limit (i.e. Portugal). As RAC was introduced into the BRT models as a variables, we needed to assign RAC values to those 5 × 5 km cells not sampled to produce a predictability map. To do this, we assigned a residual mean value for each non-sampled cell to generate a predictability estimate. Badger presence predictability for mainland Portugal was estimated using the “predict.gbm” function available in the R package “gbm”^97^, which allows estimating predicted values from generalized boosted models. A raster layer was produced based on those predicted values and using the “raster”^98^ package, which was exported to a Geographical Information System (Quantum GIS 2.14.987), where we created a predictability map.

Although producing a predictability map is important to understand species distribution and to define effective conservation plans, in regions were species presence may be more scattered or irregular (due to temporal variation of resource availability), as at the limits of a species’ distribution, predictability may be less informative. In contrast, favourability, defined by Real and colleagues^100^ as the “variation in the probability of occurrence of an event in certain conditions with respect to the overall prevalence of the event”, may be a more adequate index to assess which regions might be more adequate to support a badger population given the regional context of the species’ distribution. Since a favourability index of 0.5 indicates that cells/conditions have a probability of harbouring badgers equivalent to the overall presence of badgers in the entire dataset, the use of the favourability function allowed us to discriminate between cells that favour badger presence (F ≥ 0.50) and those that possess deleterious characteristics for badgers (F < 0.5)^101^. Thus, favourability is an important index in conservation biology and particularly for identifying routes of expansion or retraction. Favourability was estimated as described by Real and colleagues^100^ and Acevedo and Real^101^, based on the predictability results and the number of presences (*n*_1_) and number of absences (*n*_0_).

We validated the predicted performance of the favourability map by matching it with previously recorded badger presence data [obtained non-systematically from other published or unpublished studies; mostly roadkills (e.g.,^102^), captures (e.g.,^43^) and camera-trapping (e.g.,^103^) data; Fig. S2, Supplementary Material], and estimated the percentage of false negatives, i.e. cells with a favourability less than 0.50 but where badger presence was confirmed. All statistical analysis were implement using R software^104^.

Finally, we forecasted the evolution of badger distribution in Portugal up to 2040. Based on our model results, we predicted the favourability of a territory to harbour badgers based on variables with the potential for change: landcover and climate (see Results). Soil and rock types were assumed to stay unchanged. We also opted to keep constant the densities of domestic ungulates because there are no available national predictions on how these variables may evolve up to 2040.

Land-use change scenarios for 2040 were based on predictions and data provided by Stürck and colleagues (^38^; http://labs.kh.hercules-landscapes.eu/labs/themeLD.html). These authors developed four scenarios [Libertarian Europe - A1; Eurosceptic Europe - A2; Social Democracy Europe - B1; European Localism - B2; see^38^ for a detailed description of the scenarios]. As the landcover categories used in our study varied slightly from those described by Stürck and colleagues^38^, we grouped categories of their study that could be assigned according to those identified in our study as influential: Shrublands (semi-natural vegetation, recently abandoned arable land, and Heatland and moorlands^38^); Herbaceous (Pasture and recently abandoned pasture land^38^). *Eucalyptus* cover was assumed to stay constant since current Portuguese legislation prohibits an increase of these exotic plantations and wood/paper production is likely to continue.

Climate data predictions (i.e. annual precipitation and annual mean temperature; see Results) were obtained from the Intergovernmental Panel on Climate Change (IPCC) climatic dataset^17^, based on scenario A1B from the IPCC Special Report on Emission Scenarios (SRES^17^), which assumes a global economy with a balanced use of energy systems (fossil and non-fossil^105^). Climate change scenarios for Portugal estimate that in 2040 there will be a generalized reduction in annual mean precipitation (an average of 10% for the entire country^17^). Inversely, temperatures are expected to rise, with most of the country showing an average increase of 1.5 °C^17^.

Based on these predicted scenarios, we estimated the values of the variables included in the best model for every 10 × 10 km grid cell in 2040 and created a favourability map based on the assessed model parameters, using the same methodology as detailed above.

## Supplementary information

Supplementary Material

## Data Availability

All badger’s presence data will be available at the “Atlas of Portuguese mammals” website database: http://atlas-mamiferos.uevora.pt/index.php/downloads/.
