# Supplementary material for: Climate and landscape changes as driving forces for future range shift in southern populations of the European badger
Source: Sci Rep. 2019 Feb 28;9:3155. doi: 10.1038/s41598-019-39713-1 (PMC6395600; doi:10.1038/s41598-019-39713-1)
Supplement: Supplementary file 1 — Supplementary Material [file 41598_2019_39713_MOESM1_ESM.pdf]

Climate and landscape changes as driving forces for future range shift in southern populations of the European badger

Luís M. Rosalino[1,2], Diana Guedes[1], Diogo Cabecinha[2], Ana Serronha[3], Clara Grilo[1,], Margarida Santos-Reis[2], Pedro Monterroso[3], João Carvalho[1,4], Carlos Fonseca[1], Xosé Pardavila[5], Emílio Virgós[6], Dário Hipólito[1]

[1] Departamento de Biologia & CESAM, Universidade de Aveiro, 3810-193 Aveiro, Portugal;

[2] cE3c- Centre for Ecology, Evolution and Environmental Changes, Faculdade de Ciências da Universidade de Lisboa, 1749-016 Lisbon, Portugal;

[3] CIBIO/InBIO, Centro de Investigação em Biodiversidade e Recursos Genéticos, Universidade do Porto, Campus Agrário de Vairão, 4485-661 Vairão, Portugal;

[4] Wildlife Ecology & Health group (WE&H) and Servei d'Ecopatologia de Fauna Salvatge (SEFaS), Departament de Medicina i Cirurgia Animals, Universitat Autònoma de Barcelona, 08193-Bellaterra, Barcelona, Spain;

[5] Área de Ecoloxía, Departamento de Bioloxía Funcional, Universidade de Santiago de Compostela, Facultade de Bioloxía - Rúa Lope Gómez de Marzoa, s/n. Campus sur. 15782 Santiago de Compostela, Spain;

[6] ESCET, Departamento de Biología, Geología, Física y Química Inorgánica, Universidad Rey Juan Carlos, C/Tulipán s/n., 28933 Móstoles, Spain.

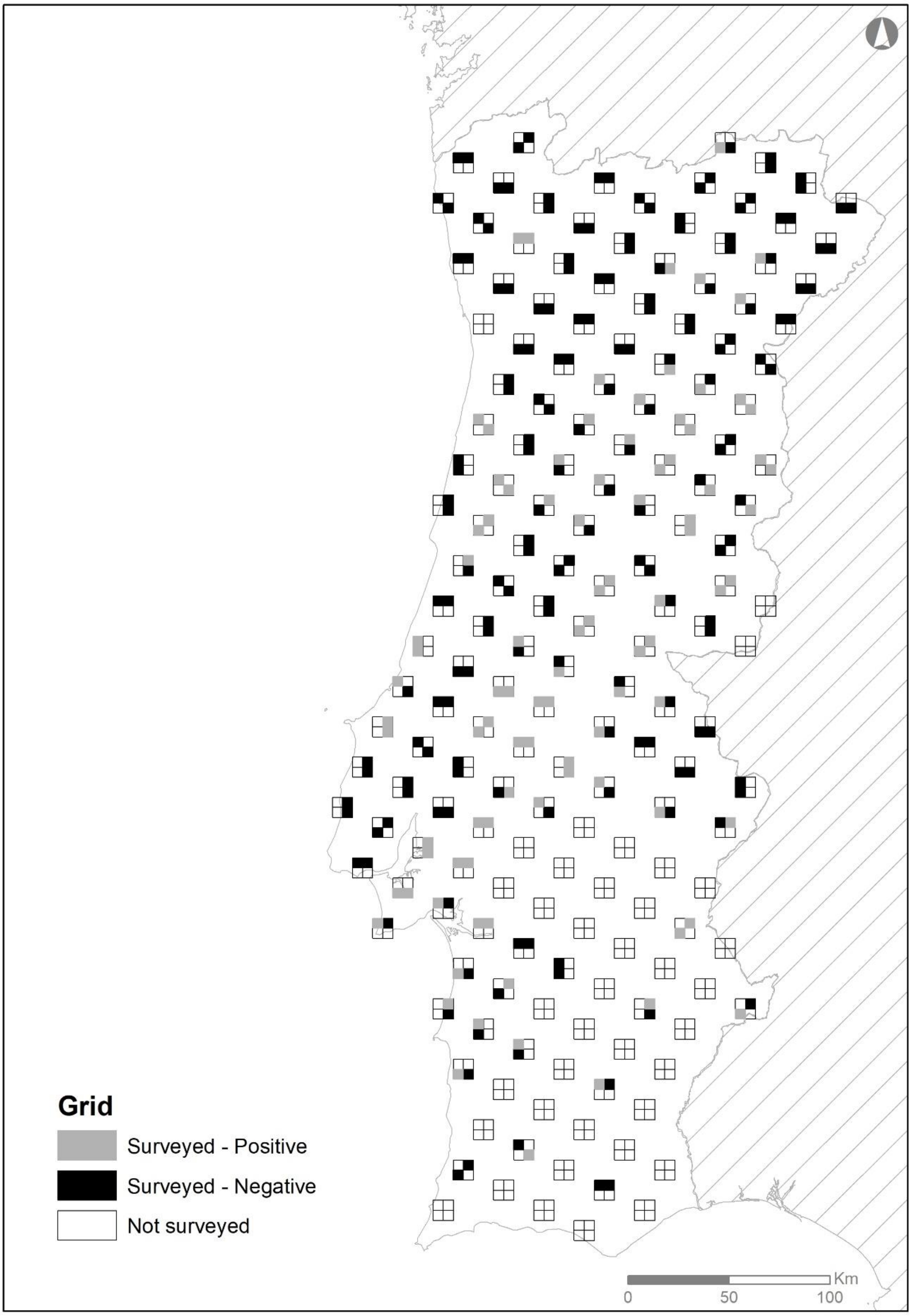

Fig S1. Sampled 5x5 km cells where signs of badger presence were detected.

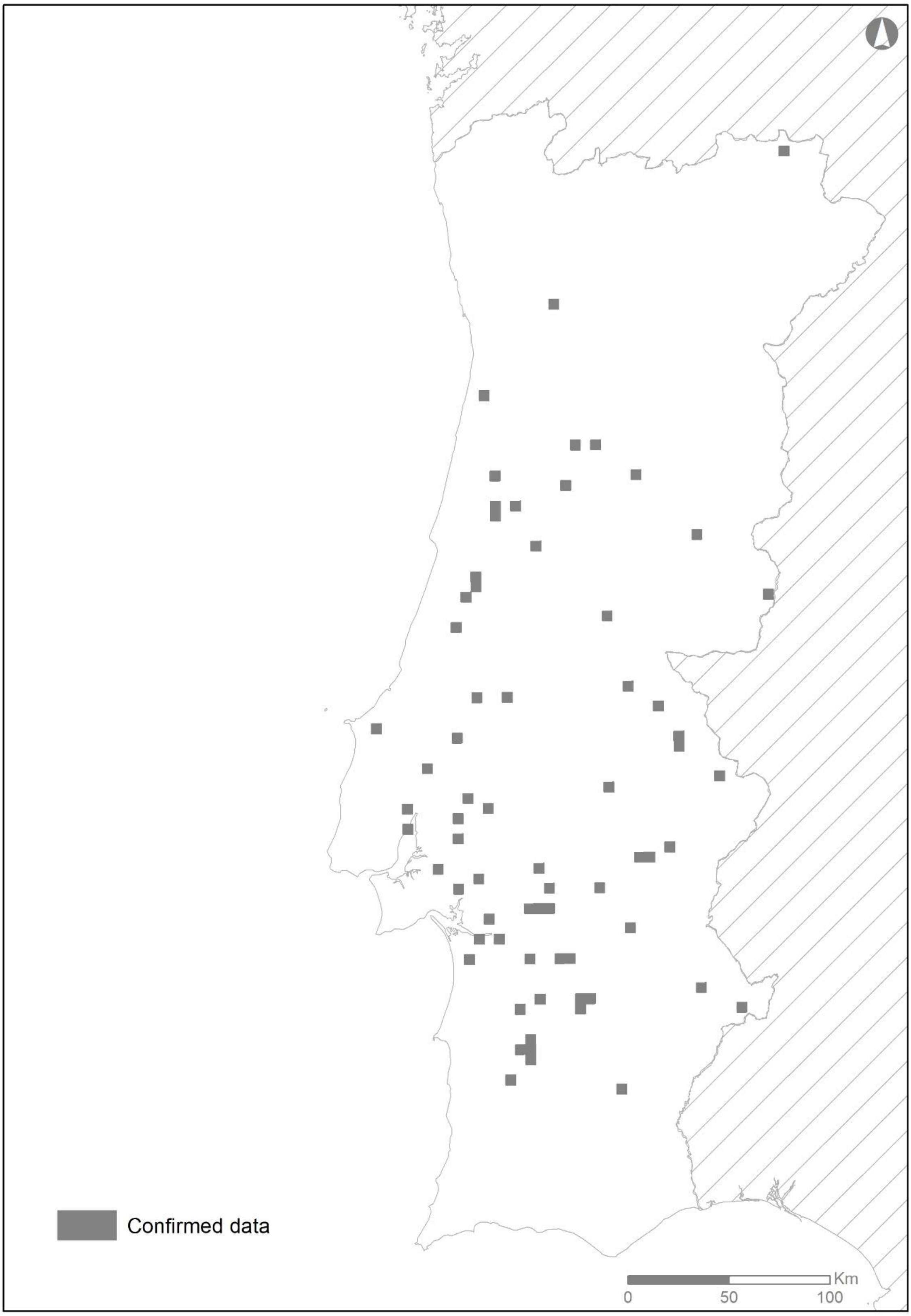

Fig S2. Distribution of the independent badger presence data used to test accuracy of the best RAC-BRT model.

Table S1 - Variance Inflation Factor (VIF) for each continuous covariates included in hypothesis H1. Variables with VIF values higher than 5 were excluded from further analysis. The VIF was recalculated for the remaining variables. The process was repeated until none of the retained variables reached VIF>5.

Variable	**VIF**
Coniferous	2.566203
Shrublands	2.504710
Food	1.965273
Herbaceous	1.947030
Eucalyptus	1.888728
Deciduous	1.842170
Agroforestry	1.821995
Artificial	1.420192
H	1.120240
Wetlands	1.106249
Exotic	1.071054

Table S2 - Variance Inflation Factor (VIF) for each continuous covariates included in hypothesis H2. Variables with VIF values higher than 5 were excluded from further analysis. The VIF was recalculated for the remaining variables. The process was repeated until none of the retained variables reached VIF>5.

**Variable**	**VIF**
Paved_roads	3.027804
Human_pop	2.471251
Highways	1.438579
Goat&sheep	1.093621
PA	1.093585
Cattle	1.074238
Unpaved_roads	1.062543
Hunting	1.049642
Pigs	1.011846

Table S3 - Variance Inflation Factor (VIF) for each continuous covariates included in hypothesis H3. Variables with VIF values higher than 5 were excluded from further analysis. The VIF was recalculated for the remaining variables. The process was repeated until none of the retained variables reached VIF>5.

**Variable**	**VIF (Initial)**	**VIF (Final)**
Sediment	47.019247	*removed
Sediment/Metamorph	44.746818	3.234831
Eruptive	39.538041	2.911627
Cambisols	4.934294	4.488028
Lithosols	3.568110	3.378787
Luvisols	2.939209	2.705969
Podzols	2.773519	2.574867
Alt_mean	2.508845	2.492825
Alt_range	2.036755	2.035352

Table S4 - Variance Inflation Factor (VIF) for each continuous covariates included in hypothesis H4. Variables with VIF values higher than 5 were excluded from further analysis. The VIF was recalculated for the remaining variables. The process was repeated until none of the retained variables reached VIF>5.

**Variable**	**VIF**
Ann_Temp	3.066682
Ann_Prec	2.190609
Prec_season	2.051523
Temp_season	1.428217

Table S5 - Variance Inflation Factor (VIF) for each continuous covariates included in hypothesis H5. Variables with VIF values higher than 5 were excluded from further analysis. The VIF was recalculated for the remaining variables. The process was repeated until none of the retained variables reached VIF>5.

**Variable**	**VIF**
Ann_Prec	3.540072
Ann_Temp	3.188764
Shrublands	1.752918
Eruptive	1.721719
Herbaceous	1.538916
Podzols	1.324292
Eucalyptus	1.270405
Cattle	1.207367
Pigs	1.116017
